# A single-blinded, randomized, parallel group superiority trial investigating the effects of footwear and custom foot orthoses versus footwear alone in individuals with patellofemoral joint osteoarthritis: a phase II pilot trial protocol

**DOI:** 10.1186/s13047-017-0200-y

**Published:** 2017-04-26

**Authors:** Narelle Wyndow, Kay M. Crossley, Bill Vicenzino, Kylie Tucker, Natalie J. Collins

**Affiliations:** 1 School of Health and Rehabilitation Sciences, The University of Queensland, St Lucia, 4072 QLD Australia; 2 School of Allied Health, College of Science, Health and Engineering, La Trobe University, Bundoora, 3086 VIC Australia; 3 School of Biomedical Sciences, The University of Queensland, St Lucia, 4072 QLD Australia

**Keywords:** Randomised controlled trial, Phase II trial, Footwear, Foot orthoses, Patellofemoral, Osteoarthritis

## Abstract

**Background:**

Patellofemoral joint osteoarthritis is a common condition, yet information regarding conservative management is lacking. Foot orthoses are an effective intervention for improving pain and function in younger individuals with patellofemoral pain and may be effective in those with patellofemoral osteoarthritis. This pilot study will seek to establish the feasibility of a phase III randomised controlled trial to investigate whether foot orthoses worn in prescribed motion controlled footwear are superior to prescribed motion control footwear alone in the management of patellofemoral osteoarthritis.

**Methods/design:**

This phase II pilot clinical trial is designed as a randomized, single-blind, parallel group, two arm, superiority trial. The trial will recruit 44 participants from Queensland and Tasmania, Australia. Volunteers aged 40 years and over must have clinical symptoms and radiographic evidence of patellofemoral osteoarthritis to be eligible for inclusion. Those eligible will be randomized to receive either foot orthoses and prescribed motion control shoes, or prescribed motion control shoes alone, to be worn for a period of 4 months. The feasibility of a phase III clinical trial will be evaluated by assessing factors such as recruitment rate, number of eligible participants, participant compliance with the study protocol, adverse events, and drop-out rate. A secondary aim of the study will be to determine completion rates and calculate effect sizes for patient reported outcome measures such as knee-related symptoms, function, quality of life, kinesiophobia, self-efficacy, general and mental health, and physical activity at 2 and 4 months. Primary outcomes will be reported descriptively while effect sizes and 95% confidence intervals will be calculated for the secondary outcome measures. Data will be analysed using an intention-to-treat principle.

**Discussion:**

The results of this pilot trial will help determine the feasibility of a phase III clinical trial investigating whether foot orthoses plus motion control footwear are superior to motion control footwear alone in individuals with patellofemoral osteoarthritis. A Phase III clinical trial will help guide footwear and foot orthoses recommendations in the clinical management of this disorder.

**Trial registration:**

Retrospectively registered with the Australian New Zealand Clinical Trials Registry: ACTRN12615000002583. Date registered: 07/01/15.

## Background

Osteoarthritis (OA) is a leading cause of musculoskeletal pain and disability worldwide [1]. Knee OA is common, affecting up to one-third of individuals aged over 60 years [2]. Individuals with knee OA experience significant pain, functional disability and poorer quality of life compared to age-matched controls [3]. Importantly, knee OA has no cure. Those with end-stage disease typically undergo total knee replacement, which is associated with annual costs exceeding $145 million per year in Australian public hospitals alone [4].

Based on radiographic and magnetic resonance imaging (MRI) evidence, the patellofemoral (PF) joint is the knee compartment most commonly affected by OA [5–7]. When considering the compartments individually, isolated PF OA is more prevalent than isolated tibiofemoral (TF) OA (PF OA: 15–25%, TF OA: 1–17%) [5–7]. Notably, even mild radiographic PF OA is associated with greater pain and functional limitations when compared to similar levels of radiographic TF OA [8]. Patellofemoral OA is observed in 55% of people aged 40 to 50 years of age who have PF pain [5] and has the potential to significantly impact on economic productivity, quality of life and daily function.

Despite the substantial individual and societal burden of PF OA, there is surprisingly little evidence for effective treatments. Based on similarities in clinical symptoms, structure and function between PF OA and PF pain in adolescents and young adults [9], it is plausible that treatments with known efficacy for PF pain may also be effective for PF OA. Support for this suggestion comes from a recent randomized controlled trial (RCT), which demonstrated that a physical therapy program developed for PF pain [10] is also effective in improving pain in older adults with PF OA [11].

Foot orthoses, inserts worn in everyday footwear, are another intervention with potential to be an effective intervention for PF OA. In those with PF pain, there is evidence from RCTs that foot orthoses are significantly more efficacious than a wait-and-see approach [12] and flat insoles [13] over 6 weeks, with few and mild adverse effects observed. We have shown that individuals with PF OA experience immediate improvements in pain when performing functional tasks with foot orthoses, compared to shoes alone [14]. While mechanisms of foot orthoses effects in PFP and OA are unclear, it has been proposed that foot orthoses may reduce the internal rotation of the tibia and femur that often accompanies foot pronation [15] thereby reducing lateral forces of the patella against the femur and resultant PF joint stress [16]. The longer-term effects of foot orthoses on pain and function in those with PF OA have yet to be evaluated.

### Objectives

The primary objective of this study is to establish the feasibility of a phase III randomized clinical trial to determine if customized foot orthoses worn in prescribed motion control footwear are superior to prescribed footwear alone, in individuals with PF OA over 4 months. Specifically, feasibility will be assessed by evaluating: (i) participant recruitment rate; (ii) the number of eligible participants; (iii) the willingness of participants to commit to the study protocol; (iv) optimal time frames for manufacture and provision of the interventions; (v) adherence to the interventions over the 4 months of the study; (vi) the number of adverse events; (vii) diary completion, and; (viii) drop-out rate.

The secondary objective of this study is to determine completion rates and calculate treatment effect sizes for patient reported outcomes assessing knee-related symptoms, function, quality of life, kinesiophobia, self-efficacy, general and mental health, physical activity, treatment satisfaction, treatment success, and self-reported recovery rate, at two and 4 months. Two measures, the change in average and worst knee pain rated on a visual analogue scale, and patient-perceived global improvement from the interventions, will form the primary outcomes for a phase III trial and will assist in sample size calculations.

## Methods

### Experimental design

This pilot study is a 4-month randomized, assessor-blind, multicenter, parallel group superiority trial with a 1:1 allocation. The trial design was developed conforming to Standard Protocol Items: Recommendations for Interventional Trials (SPIRIT) guidelines [17, 18].

### Study setting

This trial will involve recruitment of volunteers in the greater Brisbane area of Queensland, and Hobart, Tasmania (Australia). The Brisbane cohort will be assessed at The University of Queensland’s research laboratories, while the Hobart cohort will be assessed in a private podiatric practice. Both sites will utilize the same multifaceted recruitment strategy similar to our previous clinical trials [11, 13]. Paid advertisements will be placed at regular intervals in local and regional newspapers and on social media platforms. This will be accompanied by regular posting of advertisements on university, gymnasium and community noticeboards. Local health practitioners will be made aware of the study through information and advertising packages. We will also recruit from existing research databases of individuals with PF pain and OA.

Ethics approval for both sites has been granted by The University of Queensland’s Medical Research Ethics Committee (approval number: 2014000068). This ethics committee will also oversee any protocol amendments during the trial. All participants will provide informed written consent prior to participating in the study.

### Eligibility criteria

#### Inclusion criteria

Male and female volunteers will be eligible for inclusion in the study if they meet the following criteria: i) aged ≥ 40 years; ii) anterior knee pain aggravated by at least two activities that load the PF joint (e.g. squatting, stair ambulation); iii) pain during these activities present on most days in the past month; iv) pain severity ≥30 mm on a 100 mm visual analogue scale (VAS) during aggravating activities; and v) radiographic evidence of PF OA, including joint space narrowing and/or presence of osteophytes (Kellgren and Lawrence ≥ grade 1 [19]).

#### Exclusion criteria

Volunteers will be excluded if they meet any of the following criteria: (i) concomitant pain from other knee structures (including the TF joint), hip or lumbar spine; (ii) recent treatment for PF pain (knee injections within the previous 3 months; foot orthoses or physiotherapy within the previous 12 months); (iii) any foot condition precluding the use of foot orthoses; (iv) knee or hip arthroplasty or osteotomy; (v) planned lower limb surgery in the following 4 months; (vi) moderate to severe concomitant TF OA (Kellgren and Lawrence grade ≥ 3 on radiograph); (vii) neurological or systemic arthritis conditions; (viii) physical inability to undertake testing procedures; (ix) contraindications to x-ray (e.g. pregnancy, breastfeeding); or (x) inability to understand written and spoken English.

### Interventions

Allocated interventions will be administered by the primary investigator (NW), who is a podiatrist with over 25 years experience professionally registered with the Podiatry Board of Australia. The interventions will be issued 2 to 4 weeks after the initial baseline testing session. All participants will receive an educational package outlining wearing-in procedures and the importance of regular use of the interventions. Clear verbal and written instructions will be provided on how to acclimatize to the intervention over a period of 1 week, according to standard clinical protocols. For example, on the first day of use, participants will be asked to wear the allocated intervention for one hour during light activity (preferably around the home), then two hours on the second day, three hours the third day, and so on, until eight hours of continuous use is achieved. Participants are then encouraged to continue to wear the interventions for at least 8 h each day thereafter. In addition, general information on PF OA and advice regarding management of the condition will be provided. Participants will be encouraged to continue with all regular activities. We will also advise patients to avoid using additional pain medications (other than those used regularly prior to study commencement), topical preparations for their knee, knee braces, or additional physical therapies during the study period.

#### Foot orthoses

This study will utilise custom-made foot orthoses. The older cohort targeted in this trial is expected to display a greater range of foot deformity and morphologies [20] than those observed in PF pain cohorts under 40 years of age. Commencing with an individualised, neutral semi-weight bearing foot scan for the fabrication of the foot orthoses will partially address this issue [13, 21].

To fabricate the devices, 3D foot contours will be acquired during the baseline visit via an Orthema® Mobile 08/12 digitiser with Orthowin Software (version 4.80.80, Rotkreuz, Switzerland), using standardised procedures detailed in Table 1. The foot digitiser has 544 mechanical measuring pins, which elevate to obtain a full contact 3D measure of the foot contours. All scans will be taken by the same investigator (NW), who has over 6 years experience with the Orthema system. Scans will be acquired according to a standardised protocol (Table 1) similar to the gait-referenced system used by Trotter et al. [22, 23] to acquire foam impressions of the foot.

**Table 1:** Foot scanning protocol

Foot scanning protocol	
1	One foot is placed in a semi-weight bearing position with the other foot on the floor for stability.
2	Foot posture is adjusted to obtain a neutral talonavicular position determined by manual palpation, with the supra- and infra-malleolar curves of equivalent curvature [24].
3	The long axis of the foot is aligned in the sagittal plane with the long axis of the scanner plate.
4	The hip, knee and ankle joints are aligned in the sagittal plane.
5	The medial forefoot is loaded to contact the scanning plate if possible, to correct for soft tissue forefoot varus alignment. In the presence of a rigid forefoot varus deformity, the forefoot will be medially loaded until firm resistance is met, taking care to not negatively affect rearfoot posture.
6	Tibialis anterior activation will be monitored visually to ensure minimal active forefoot distortion.

**Fig. 1:**
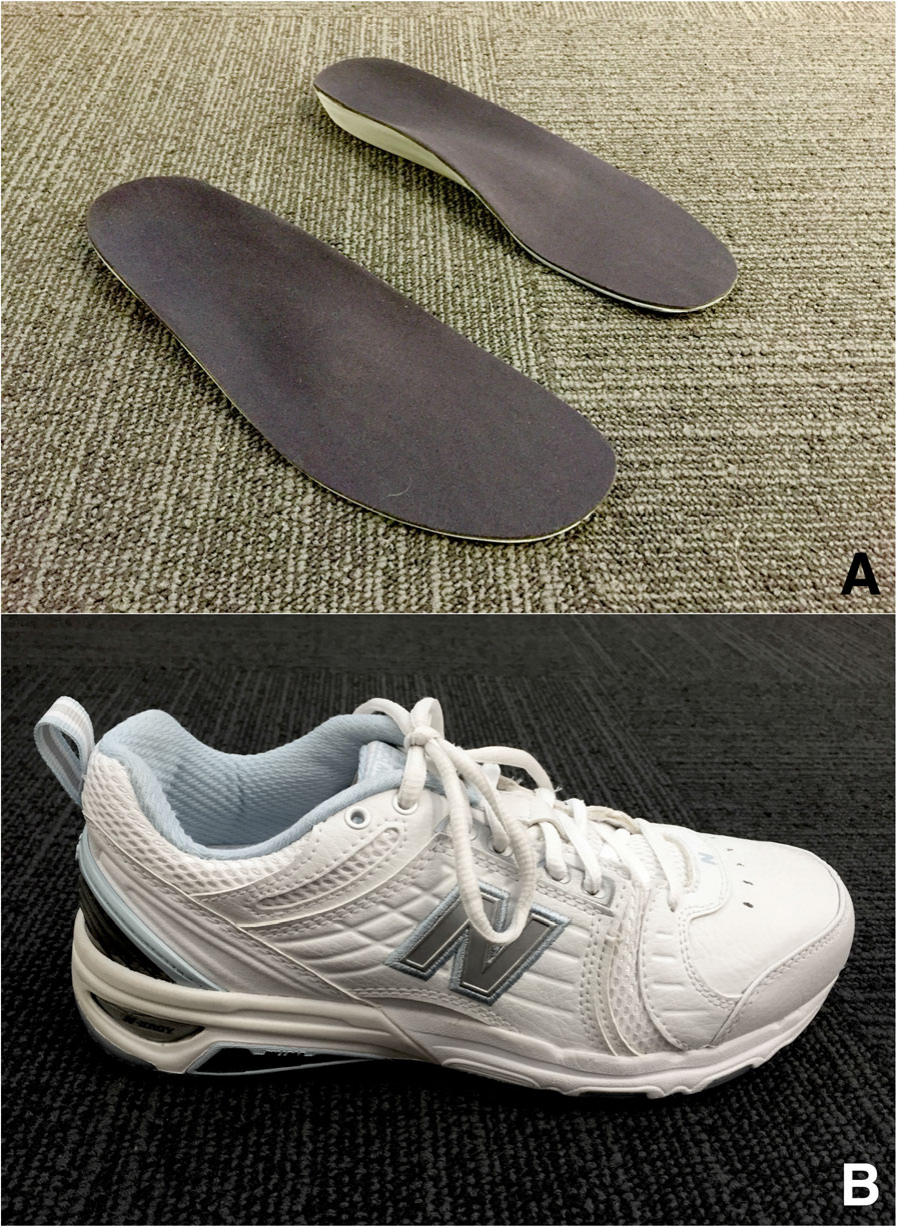
**a** Customized EVA foot orthoses. **b** New Balance 857 motion control cross-trainer

Foot orthoses will be modified using Orthema Orthowin Software (version 4.80.80, Rotkreuz, Switzerland). Additions will be according to a standardized procedure (Table 2). All participants will receive a metatarsal dome, cuboid pad, heel cup contour and a plantar fascia groove. A medial or lateral rear-midfoot wedge will be added, depending on the degree of foot pronation or supination rated using the Foot Posture Index [24], and the degree of pronation or supination (mild, moderate, severe) observed upon visual gait assessment, reflecting clinical practice. The orthoses will be fabricated by Orthema Australasia (Brisbane, Australia) from single medium to high density EVA bases (Shore A 45°). The orthoses will be milled in an Orthema® M65 CNC Milling Machine (Rotkreuz, Switzerland) into a size-matched orthotic blank. The rough milled device will be undercut in the arch to improve shoe fit, then covered in 1.5 mm medical grade neoprene (OrthoNeo, Orthema Australasia) and buffed (Fig. 1a).

**Table 2:** Orthema Orthowin® Software Addition Protocol and Orthotic Fitting Modifications

CAD software adjustments		
	Addition type	Description
1.	Heel cup contour	▪ Width of horseshoe shape adjusted to create smooth heel contour
2.	Medial rearfoot skive	▪ Height dependant on degree of pronation as rated by the FPI: Mild: 140% (height approx. 23 mm; FPI: 0–4), Moderate: 170% (height approx. 28 mm; FPI: 5–8), Severe: 200% (height approx. 33 mm; FPI: 9+) ▪ No medial skive will be added to a supinated foot type
3.	Cuboid pad	▪ 50% height (approx. 10 mm) ▪ 150% of original addition length added to region of cuboid (approx. 122 mm)
4.	Metatarsal dome	▪ Standard shape at 90% height (approx. 5 mm) and width adjusted to leave approx. 1.5 cm either side of block ▪ Length adjusted to extend from proximal metatarsal heads to base of metatarsals
5.	Rearfoot-midfoot supination/pronation wedge	▪ Height adjusted dependent on degree of pronation or supination as rated by the FPI Mild: 140% (height approx. 13 mm; FPI: 0–4), Moderate: 170% (height approx. 17 mm; FPI: 5–8), Severe: 200% (height approx. 20 mm; FPI: 9+) ▪ Length adjusted to finish just proximal to 1^st^ metatarsal head
6.	Plantar fascia groove	▪ 2 mm depth ▪ Length adjusted to span medial longitudinal arch along approx. line of plantar fascia and the FHL tendon

*Abbreviations*: *CAD* computer assisted design, *approx.* approximately, *cm* centimeters, *FHL* flexor hallucis longus, *mm* millimeters, *FPI* Foot Posture Index. Height in mm from zero point of milling plate to the top of the adjustment

#### Footwear

All participants in both groups will receive one pair of standardized shoes. Those allocated to the foot orthoses intervention group will wear their orthoses in the prescribed shoe. We chose to prescribe a standardized shoe for participants to wear the orthoses in, on the basis that good quality footwear alone exerts therapeutic effects on lower limb pain and function [25], and has the potential to alter lower limb kinematics at the knee [26]. Thus, footwear may influence the therapeutic effects of foot orthoses. It has been recommended that footwear be controlled for in studies investigating clinical outcomes in knee pain populations [27, 28]. Participants allocated to the footwear intervention will use the prescribed shoes alone for the study duration.

Footwear will be sized by an independent professional retailer (The Athletes Foot, Brisbane, Queensland; The Running Edge, Hobart, Tasmania) approximately 2 weeks after baseline testing, and sent directly to the primary investigator (NW) who will administer them to participants approximately 4 weeks after baseline testing. If the participant is allocated to the foot orthoses group, they will also collect their foot orthoses at the time the footwear is issued. Participants will receive a New Balance 857 cross-trainer (Boston, Massachusetts) (Fig. 1b). This shoe is a neutral motion control cross-trainer, available in multiple width fittings to accommodate most foot dimensions. It features medial and lateral thermoplastic urethane posts so as to not induce a medial or lateral bias to rear- and mid-foot alignment. The posts are further reinforced by a graphite ROLLBAR® for motion control. The midsole of the New Balance 857 is dual density. The midsole density at the forefoot is 57 ± 3 Asker C (equivalent to Shore A 35°), while the heel is 65 ± 3 Asker C (equivalent to Shore A 40°). These densities exceed minimum density requirements to reduce falls risk in older populations (Shore A 33°) [29]. Due to these features, the shoe does not compress easily and will maintain the prescribed wedging included in the orthoses, in addition to ameliorating any increased risk of falls in this older cohort.

#### Modifications to interventions

**Fig. 2:**
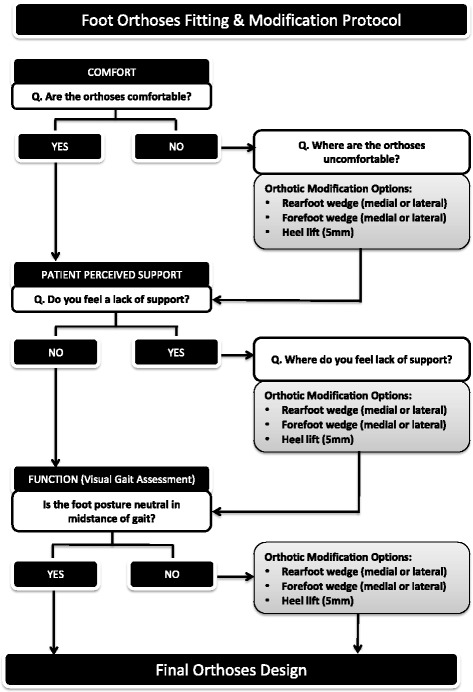
Foot orthoses fitting and modification protocol

The orthoses may be modified at the initial fitting session to optimize comfort and foot alignment when worn in the prescribed footwear. Modifications will be according to a standardized protocol based on our previous RCT of foot orthoses for PF pain [13] (see Fig. 2). If modifications are required, the orthoses will initially be modified for comfort using self-adhesive wedges (Formthotic® firm 6 mm extended wedges, Christchurch New Zealand). Once comfort has been achieved, the effect of the orthoses on the support of the foot alignment during gait will be visually determined. The orthoses may be further modified using the same steps described in Fig. 2 to attain a more neutral rear and midfoot posture as required.

Footwear may only be modified by superficial adjustments to improve comfort. Examples include changing lacing configuration, stretching the leather upper over prominent digital deformities, or internally padding seams that may rub on bony prominences. All modifications will be kept to a minimum and fully documented by the primary investigator.

While inclusion/exclusion criteria aim to ensure that participants do not exhibit other significant lower limb pathology, it is possible that either intervention may generate new pain in addition to exacerbating existing knee pain. If new or increased pain occurs, and the interventions are suspected to be the cause, this will be managed using standard clinical practice principles. The interventions will be withdrawn until the new pain settles, and slowly reinstated as able and without exacerbating any pain. Should the pain return, the treatment will be ceased. All increases in pain will be reported as an adverse event. Participants will be questioned regarding any adverse effects from either intervention at 3 weeks, 8 weeks and at the completion of the study (16 weeks).

### Adherence & retention

To encourage adherence, when interventions are issued at baseline, all participants will be informed that both interventions are ‘active’ interventions i.e. neither is a control intervention. Those in the footwear group will be informed of the beneficial nature of good footwear on knee pain and function in those with OA in other regions of the knee, to reduce resentful demoralization if not allocated to the foot orthoses and footwear group.

#### Diary

For the 4-month duration of the study, participants will be asked to keep a daily log of hours of footwear use; hours of foot orthoses use (if applicable); hours of use of alternate footwear (including description of footwear style); knee pain severity (rated on a 10-point numerical rating scale); medications used; and a description of general activities undertaken.

#### Phone and physical review

Three weeks after the provision of the interventions, all participants will be contacted via phone or email for follow-up regarding their allocated intervention. If participants have any concerns about their intervention, they will be invited for review the following week (week 4) in person with the primary investigator (NW) to ensure optimal comfort and function. If participants experience adverse effects (e.g. excessive pressure from the foot orthoses, blistering, increase in knee pain), modifications will be made by the primary investigator. Participants experiencing difficulties will be contacted by phone or email at weekly intervals thereafter to ensure they are progressing satisfactorily until they are comfortable with their intervention. All participants will be contacted via email at 8 weeks regarding their allocated intervention (at 2 month follow-up). If participants report no ongoing problems with their allocated intervention, they will not be contacted again until the completion of the study, as is common in clinical settings.

### Concomitant care

Participants will be permitted to continue use of their normal medications, including anti-inflammatory medications they had been taking on a regular basis prior to commencing the study. They will be discouraged from increasing the dose of any usual pain medication without informing the investigators. Participants will be encouraged to refrain from using any additional physical therapies or other orthotic devices (e.g. knee supports) for the duration of the study, and to contact the primary investigator if they are not satisfied with their allocated intervention. Participants will be asked at 2- and 4-month follow-up to provide details of any concomitant interventions.

### Outcome measures

All participants will complete a battery of patient reported outcome measures at baseline, 2 and 4 months. Should more than 2 months elapse since completion of the baseline questionnaires and the issuing of trial interventions, then a second ‘delayed baseline’ questionnaire battery will be completed. This will ensure an accurate baseline assessment, as well as assessment of the stability of the measurement tools in the target population. Data collection will be via a web-based platform or paper version, based on participant preference, although participants will be encouraged to utilize the web-based platform where possible. Baseline demographic data will include age, sex, weight, height, occupation and employment status, affected knee, symptom duration, and self-reported presence of knee crepitus. The 4-month outcomes will be the primary end point.

#### Primary outcome measures

The primary outcome of this study will be to determine the feasibility of a full-scale randomized controlled trial. Key outcomes to assess feasibility will include: (i) evaluating recruitment rate as the percentage of eligible participants of those who made initial contact to determine if current eligibility criteria are too open or restrictive; (ii) the willingness of participants to commit to the study protocol at the recruitment phase; (iii) adherence to the interventions over 4 months and whether this varies between groups; (iv) to determine optimal data collection methods for patient reported outcomes; (v) the number of adverse events reported in each group over 4 months; (vi) diary completion, and; (vii) drop-out rate.

In addition, optimal time frames for manufacture and provision of the interventions at the start of the pilot trial will be determined to enable streamlining of provision for the phase III trial.

#### Secondary outcome measures

Secondary outcome measures for this pilot trial will focus on completion rates and missing data of patient reported outcomes to determine those to be utilized in the phase III trial. Patient reported outcomes will be administered at baseline, 2 and 4 months, and will evaluate dimensions of pain, symptoms, function and physical activity level, quality of life, kinesiophobia, self-efficacy, and general and mental health. In addition, five questions pertaining to treatment outcomes will be completed at 2 and 4 months (Questions 2–6 below).

1. *Knee pain severity* will be evaluated at baseline, 2 and 4 months on a 100 mm visual analogue scale anchored with no distress (0) and unbearable distress (10). Usual pain on movement in the previous week and worst knee pain during a nominated aggravating activity in the previous week will be assessed [30]. The VAS for usual and worst pain is reliable, valid and responsive to change in individuals with PF pain, with a change of 20 mm representing the minimal clinically importance difference (MCID) [30].
2. *Global rating of change* (GROC) will be evaluated at 2- and 4-month follow-up. Participants will be asked to rate their overall change in knee pain on a six-point Likert scale (completely recovered, much improved, improved, no change, worse, much worse). GROC has been used to evaluate outcomes in large clinical trials of PF pain and OA [10, 11, 13, 31].
3. *Satisfaction of treatment*. To rate participants’ satisfaction with treatment at 2 and 4 months, they will be asked to respond to the following questions using a 5-point Likert scale (very satisfied, somewhat satisfied, neither satisfied nor dissatisfied, somewhat dissatisfied, very dissatisfied):
    1. *“Over the course of treatment for your knee pain, how satisfied were you with your overall treatment?”*
    2. *“If you had to live with the symptoms you have right now, how would you feel about it?”*
4. *Success of Treatment*. Participants will be asked two questions related to their perception of treatment success at 2 and 4 months:
    1. *“Overall, would you agree that the treatment you have received has been successful for your knee pain?”* (Yes/No)
    2. *“If a good friend has the same knee pain as you, would you recommend the same treatment you received?”* (Yes/No)
5. *Self-Reported Rate of Recovery.* At 2 and 4 months, participants will be asked to provide a number from 0–100, in response to the following question:
*“On a scale of 0 (not at all) to 100% (totally recovered), how well do you feel you have recovered from your knee pain? “*
6. *Patient Acceptable Symptoms State (PASS).* The PASS will be used to assess the response to treatment by assessing the concept of wellbeing or remission of symptoms (feeling good) [32]. The PASS has been proposed to be a more appropriate measure than whether the participant has improved (feeling better) as it less sensitive to baseline levels of symptoms and better reflects what is important to participants [33]. At 2 and 4 months, the participants will be asked:
*“Is your current condition satisfactory, when you take your general functioning and your current pain into consideration?” (Yes/No)*
7. *Pain Severity and Activity Restriction*. Participants will rate their knee pain severity and activity restriction over the previous week by responding to a series of 100 mm visual analogue scales. Pain severity will be rated from 0 (no pain) to 10 (worst pain possible), in terms of average pain, worst pain, pain at rest, and pain on general movement during specific tasks (walking, sitting for one hour, rising from sitting, going up and down stairs, squatting and running). Participants will rate the average amount of restriction to their daily activities that they have experienced over the previous week due to their knee pain (0 = no restriction; 10 = maximum restriction possible). Participants also will be asked to nominate the activity which caused the most knee pain in the last week.
8. *Anterior Knee Pain Scale (AKPS)*. The AKPS is a 13-item questionnaire with discrete categories related to current knee symptoms and function [34]. Responses within each item are weighted and summed to provide an overall index, where 0 represents maximal disability and 100 represents no disability. The AKPS is reliable and valid in PF pain populations, with an MCID of 8–10 points [30].
9. *Knee Osteoarthritis Outcome Score (KOOS)*. The KOOS is a self-administered questionnaire that assesses five domains: pain, symptoms, activities of daily living function, sport and recreation function, and knee-related quality of life [35]. A 5-point Likert scale is used to score items from 0 (no problems) to 4 (extreme problems). Scores are transformed to a 0–100 scale, with zero representing extreme knee problems and 100 representing no knee problems. The KOOS has been shown to be reliable, valid and responsive to changes in pain in individuals with knee OA [36].
10. *PainDETECT*. The painDETECT questionnaire will be used to identify the presence of neuropathic pain [37]. This questionnaire consists of nine items; seven evaluating pain quality, one evaluating pain pattern, and one evaluating pain radiation. These items contribute to an aggregate score ranging from −1 to 38, where a higher score indicates more neuropathic—like symptoms. In knee OA populations, painDETECT score is associated with pressure pain thresholds, and may reflect central pain processing in this population [38].
11. *International Physical Activity Questionnaire (IPAQ)*. The IPAQ will be used to give an estimate of physical activity level across the study period. The short-form IPAQ is a seven-item questionnaire that assesses the intensity and duration of physical activity over the last 7 days [39]. Questions 1–4 ask the participant to indicate the number of days they undertook vigorous and moderate activity for a minimum of 10 min. They are also asked to rate the total number of minutes they usually undertook these activities per day. Questions 5 & 6 rate the number of days and minutes per day spent walking. Question 7 asks about the total number of days and time spent sitting. The IPAQ will be scored as a continuous measure expressed as metabolic equivalent of task (MET) minutes per week [40].
12. *Medical Outcomes Study 36-Item Short-Form Health Survey (SF-36)*. This questionnaire is a generic measure of health-related quality of life [41], and has been previously used in people with PF pain [10, 13]. Thirty-six items are used to calculate eight scores across the following domains: physical functioning, role limitations due to physical health problems, bodily pain, general health, vitality (energy/fatigue), social functioning, role limitations due to emotional problems, and mental health. Transformed scores range from 0 (worst health state) to 100 (best health state).
13. *EQ-5D*. Quality of life will be measured using the EQ-5D (EuroQoL) questionnaire [42]. The EQ-5D is a generic measure of health-related quality of life and has 5 dimensions: (i) mobility; (ii) self-care; (iii) usual activities; (iv) pain/discomfort; and (v) anxiety/depression. Each question has three possible responses: no problems, some problems, or extreme problems. The EQ-5D also has a health state scale which asks participants to rate their state of health on a 100-point scale, with 0 indicating worst imaginable health state and 100 indicating best imaginable health state. The EQ-5D has been shown to be valid and responsive to changes in individuals with PF pain [43].
14. *Tampa Scale for Kinesiophobia*. Psychological factors are associated with impairment in knee osteoarthritis [44]. Kinesiophobia, defined as “an irrational and debilitating fear of physical movement and activity resulting from a feeling of vulnerability to painful injury or (re) injury “[45] will be assessed using the Tampa Scale for Kinesiophobia. This scale consists of 17 statements on the subjective experience of injury and physical activity, which are each scored on a 4-point Likert scale ranging from “strongly disagree” to “strongly agree”. A total score from 17 to 68 is then calculated by summing the individual item scores after inversion of the individual scores of items 4, 8, 12 and 16. A score of 17 represents no fear of movement or re-injury, and 68 indicates a greater fear. The 17-item Tampa Scale for Kinesiophobia (English version) possesses a high degree of internal consistency and is predictive of disability levels after controlling for clinical pain in back and neck pain populations [46]. The Tampa Scale has been applied to knee OA populations for rating ‘activity avoidance’ and ‘somatic focus’ (the tendency to notice and report physical symptoms) [47].
15. *Arthritis Self-Efficacy Scale*. The Arthritis Self Efficacy scale will be used to measure arthritis-specific beliefs of the participants to perform specific tasks or behaviors to cope with the consequences of chronic arthritis [48, 49]. It is a reliable and widely used 20-item scale with 3 subscales: self-efficacy for managing pain (5 items); self-efficacy for physical function (9 items); and self-efficacy for controlling other symptoms (6 items) [48, 49]. Items are rated on a 10-point scale regarding certainty of their ability to perform a task, manage their pain or control their symptoms, with 0 being very uncertain and 10 being very certain. Each subscale is scored separately by taking the mean score of the subscale items. Higher scores indicate higher self-efficacy.
16. *Hospital Anxiety and Depression (HAD) Scale*. The HAD scale is a 14-item scale that will be used to investigate whether there is an association between PF OA and emotional state [50]. Seven questions relate to anxiety and seven to depression, with participants asked to select the best of four possible responses. The questions are scored from 0 to 3 with scores for anxiety and depression summed separately to give total scores for each component. Total scores of 0–7 represent no anxiety or depression, 8–10 is borderline, and 11–21 indicates the presence of an anxious or depressive state [50]. The HAD has been shown to be a reliable and valid indicator of anxiety and depression severity [50], and has been used in a previous study of physiotherapy in PF pain [51].

### Procedure

**Fig. 3:**
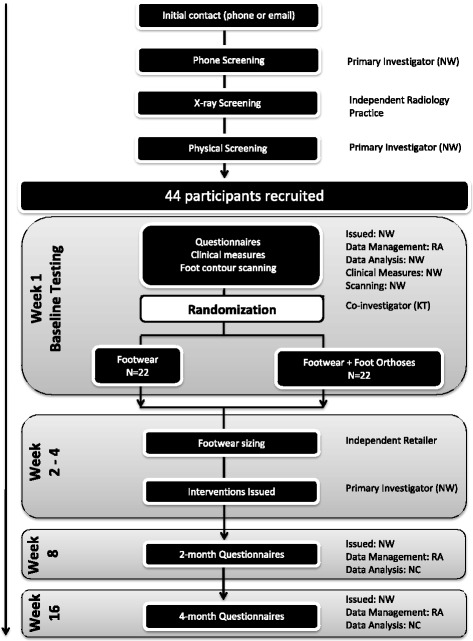
Participant flow chart. Legend NW: Narelle Wyndow; RA: research assistant; KT: Kylie Tucker; NC: Natalie Collins

Figure 3 outlines the flow of participants through the trial. Volunteers will respond to advertisements or word-of-mouth referrals by contacting the primary investigator (NW), who will also contact potential participants from existing databases via telephone, email or standard post. A three-stage screening process will be used to determine eligibility. Firstly, volunteers will be screened for major inclusion and exclusion criteria via telephone interview. Potential participants will then be invited to attend a private radiology practice to undergo knee x-rays (anteroposterior and skyline views) of their most affected knee. Volunteers who fulfill radiographic eligibility criteria will then be invited to undergo a clinical examination by a registered Podiatrist (NW) at The University of Queensland (Brisbane) or at a private podiatry practice (Hobart, Tasmania) to confirm eligibility. Suitable volunteers will provide written informed consent prior to the collection of baseline measures.

### Blinding and data collection

After baseline measures have been obtained, participants will be randomized with a 1:1 allocation to one of two interventions: i) customized foot orthoses plus footwear; or ii) footwear alone. The randomization schedule will be generated via a simple random number table by an investigator not involved in determining eligibility or in baseline, 2- or 4-month data collection procedures (KT). Allocation concealment will be maintained by this same investigator (KT) who will only reveal group allocation via phone to the primary investigator (NW) once baseline measures have been obtained.

Patient reported outcome measures will be issued either electronically or via mail by the primary investigator (NW) at baseline prior to randomization, and at 2 and 4 months. All outcome measures will be collected by a research assistant and analyzed by an investigator who will remain blinded to group allocation (NJC). Due to the nature of the interventions, neither the primary investigator providing the interventions nor the participants can be blinded to treatment group allocation. In the case of an adverse event requiring consideration of participant withdrawal, investigators who are not involved in data management and analysis will be consulted (KMC, BV).

### Sample size considerations

As this is an exploratory pilot study, sample size has been chosen based on feasibility with respect to time and funding. As such, 22 participants per group (allowing for a drop out rate of 10%) will aim to be recruited, giving an overall total of 44 participants. A full size trial requires a total of 88 participants (44 in each group allowing for 10% drop out).

### Data management and monitoring

All participant data will be coded by a research assistant, with no group identifier to maintain blinding of the investigator responsible for data analysis (NC).

Participants will be contacted regularly throughout the 4-month trial period to minimize missing data. All cases of non-adherence and non-retention will be electronically documented on a master spreadsheet to ensure appropriate handling and interpretation of results.

All electronic data will be de-identified and stored on a password-protected computer. All data in hard copy will be de-identified and kept in a locked filing cabinet in a secured office. Paper-completed outcome measures will be entered into electronic spreadsheets by the unblinded research assistant at a central site (Hobart, Tasmania).

### Planned statistical analysis

The primary feasibility outcomes for this study will be reported descriptively (e.g. %, mean (standard deviation)) for recruitment rate, number of eligible participants, drop-out rate, adherence to interventions, number of adverse events, and diary completion.

For secondary outcome measures, treatment effect sizes with 95% confidence intervals will be calculated to compare change between groups at 2 and 4 months. The *a priori* time of primary interest is 4 months. The end point for each participant will be at the completion of the study at 4 months. The suitability of each secondary outcome measures for use in the population will be assessed by the number of completed questionnaires and missing data at each time point.

Analyses will be conducted on a blinded, intention-to-treat basis where all participants randomized will be included. No data substitution will be applied to adverse event data. Analyses will be conducted under the guidance of a statistician. SPSS software (Version 23, SPSS Inv, Chicago, IL, USA) will be used to perform all analyses.

### Ancillary and post trial care

If participants experience an adverse event during the study, the trial investigators will arrange ancillary care with appropriate care providers. At the completion of the study, participants will be entitled to keep their allocated interventions. Participants who wish to obtain additional foot orthoses will be provided with a referral to a suitably qualified podiatrist, who will be provided with details of study foot orthoses design to facilitate replication of clinical benefits. All participants will be given a post-trial care pack, which details additional options for footwear and management of PF OA, such as physiotherapy. The primary investigator will be available via phone or email contact for any general enquiries regarding footwear or additional care post-trial.

### Dissemination of results

Results of this pilot trial, regardless of magnitude or direction of effect, will be presented in peer-reviewed journal articles and presented at national and international conferences. All participants will be provided with a summary of trial results via email or post.

## Discussion

Clinical guidelines prioritise conservative interventions as a first line treatment in knee OA management [52, 53]. As PF OA and PF pain in younger individuals may form a disease continuum [9], non-surgical interventions known to be effective in younger individuals with PF pain may also improve symptoms associated with PF OA. This has been demonstrated with multimodal physiotherapy [10]. Thus, it is plausible that foot orthoses, another effective intervention in PF pain [13], will also be effective in older people with PF OA [11].

This pilot trial protocol has been designed to optimize its scientific rigor by following SPIRIT guidelines and with reference to the CONSORT statement for pilot and feasibility trials [17, 18, 54]. Strengths of the trial design include randomized, concealed allocation of interventions, blinded data analysis, and the use of outcome measures with proven reliability and validity in PF pain, PF OA or general knee OA. Furthermore, we have selected interventions and outcome measures that are easily administered in a clinical setting, which improves the clinical applicability of study findings.

We have chosen to evaluate the effectiveness of customized foot orthoses for PF OA. This is on the basis that the older cohort targeted in this trial is expected to display a greater range of foot deformity and morphologies [20], which can be more readily accommodated with a customized orthoses base. This is in contrast to previous studies in PF pain in younger adults, which used prefabricated foot orthoses based on their cost effectiveness, clinical time constraints with regards to provision of devices, ease of standardization for a research setting, and wide availability [13]. However, increasing availability of computer-assisted design and manufacture of customized foot orthoses allows for more standardized adjustment of custom molded devices suitable for a research setting, as well as faster manufacturing times. This pilot trial will assist in determining time frames for provision of customized devices. Should point estimates of effects suggest positive short-term benefits of customized foot orthoses for PF OA, this trial will inform the conduct of larger RCTs with longer-term follow-up reflecting standard clinical practice (e.g. at least 12 months), and concurrent cost-benefit analyses.

Eligibility criteria for this trial do not require participants to have a particular foot type. While foot orthoses have traditionally been prescribed for conditions associated with excessive foot pronation, they have been shown to be effective in a range of foot types and conditions [13, 55, 56]. While the mechanism of effect of foot orthoses in PF pain is unclear, foot mobility rather than foot posture has better predicted outcomes with foot orthoses [57]. It is unknown as to what foot characteristics are associated with PF OA, and whether specific foot characteristics will predict outcome with foot orthoses. A recent review highlighted that with advancing age there is a decrease in foot mobility, strength and function [58]. Thus, the relationship between foot characteristics and foot orthoses outcomes in PF OA are likely to be different to younger individuals with PF pain. While this issue would benefit from investigation, individual differences in foot structure and mobility in this older cohort will be accounted for in this study by the use of customized foot orthoses.

Foot orthoses must be worn in footwear. Recent investigations into the effects of different footwear on pain and medial knee loads in individuals with medial TF OA concluded that personal footwear might have significant, potentially negative effects on knee loading and pain [28, 59]. Footwear features such as midsole density or flexibility alter the interface between the foot and the support surface, affecting both the proprioceptive input and structural support of the foot [29]. Clinical trials investigating the effect of foot orthoses on lower limb pathology must therefore consider the influence of the type of footwear worn on the stuctural support of the foot and the effect of the footwear on proprioception, as footwear has a significant effect on balance parameters and falls risk [29]. The decision to use the New Balance 857® motion control cross-trainer with foot orthoses in this study was based on its structural features, which were considered unlikely to influence the camber of the orthoses, while physically supporting the foot and providing a stable base of support to reduce risk of falls. By controlling for footwear, and using footwear as a comparator, this trial will be able to evaluate the effectiveness of customized foot orthoses, while reducing the potential influence of confounders such as footwear. This pilot trial will assist in determining participant compliance with footwear restrictions over 4 months to inform feasibility of footwear restrictions for longer term studies.

## Conclusion

This phase II pilot randomized controlled clinical trial will explore the effectiveness of foot orthoses and prescribed motion control footwear, compared to prescribed footwear alone, in the management of PF OA over a 4-month period. The information derived from this trial protocol will help inform future large-scale clinical trials on non-surgical management of PF OA.

### Trial status

Recruitment of participants commenced in November 2014, and final results are expected to be available mid 2017.

## Abbreviations

AKPS: Anterior knee pain scale
ANOVA: Analysis of variance
Approx: Approximate
EQ-5D: EuroQol
GROC: Global Rating of Change
HAD: Hospital Anxiety and Depression Scale
IPAQ: International Physical Activity Questionnaire
KOOS: Knee osteoarthritis outcome score
mm: Millimeters
n: Number
OA: Osteoarthritis
PASS: Patient acceptable symptoms state
PF: Patellofemoral
RCT: Randomized controlled trial
SF-36: Medical Outcomes Study 36-Item Short-Form Health Survey
SPIRIT: Standard Protocol Items: Recommendations for Intervention Trials
TF: Tibiofemoral
VAS: Visual analogue scale
